# Supplementary material for: Plant-derived Pembrolizumab in conjugation with IL-15Rα-IL-15 complex shows effective anti-tumor activity
Source: PLoS One. 2025 Jan 14;20(1):e0316790. doi: 10.1371/journal.pone.0316790 (PMC11731737; doi:10.1371/journal.pone.0316790)
Supplement: S1 Table — (DOCX) [file pone.0316790.s001.docx]

**S1** **Table.** Details of pembrolizumab-IL-15Rα-IL-15 sequence.

| **Components** | **Accession no.** | **Amino acid sequence** |
| --- | --- | --- |
| **Light chain** | | |
| Signal peptide | P01750 | MGWSCIILFLVATATGVHS |
| Part of restriction enzyme | - | DVQLLE |
| Pembrolizumab light chain | DB09037 | EIVLTQSPATLSLSPGERATLSCRASKGVSTSGYSYLHWYQQKPGQAPRLLIYLASYLESGVPARFSGSGSGTDFTLTISSLEPEDFAVYYCQHSRDLPLTFGGGTKVEIKRTVAAPSVFIFPPSDEQLKSGTASVVCLLNNFYPREAKVQWKVDNALQSGNSQESVTEQDSKDSTYSLSSTLTLSKADYEKHKVYACEVTHQGLSSPVTKSFNRGEC |
| **Heavy chain** | | |
| Signal peptide | P01750 | MGWSCIILFLVATATGVHS |
| Part of restriction enzyme | - | DVQLLE |
| Pembrolizumab heavy chain | DB09037 | QVQLVQSGVEVKKPGASVKVSCKASGYTFTNYYMYWVRQAPGQGLEWMGGINPSNGGTNFNEKFKNRVTLTTDSSTTTAYMELKSLQFDDTAVYYCARRDYRFDMGFDYWGQGTTVTVSSASTKGPSVFPLAPCSRSTSESTAALGCLVKDYFPEPVTVSWNSGALTSGVHTFPAVLQSSGLYSLSSVVTVPSSSLGTKTYTCNVDHKPSNTKVDKRVESKYGPPCPPCPAPEFLGGPSVFLFPPKPKDTLMISRTPEVTCVVVDVSQEDPEVQFNWYVDGVEVHNAKTKPREEQFNSTYRVVSVLTVLHQDWLNGKEYKCKVSNKGLPSSIEKTISKAKGQPREPQVYTLPPSQEEMTKNQVSLTCLVKGFYPSDIAVEWESNGQPENNYKTTPPVLDSDGSFFLYSRLTVDKSRWQEGNVFSCSVMHEALHNHYTQKSLSLSLGK |
| Linker 1 | - | GGGGSGGGGSGGGGS |
| Sushi domain of human IL-15Rα | Q13261 | ITCPPPMSVEHADIWVKSYSLYSRERYICNSGFKRKAGTSSLTECVLNKATNVAHWTTPSLKCIR |
| Linker 2 | - | GGGGSGGGGSGGGGSGGGGS |
| Human IL-15 | P40933 | NWVNVISDLKKIEDLIQSMHIDATLYTESDVHPSCKVTAMKCFLLELQVISLESGDASIHDTVENLIILANNSLSSNGNVTESGCKECEELEEKNIKEFLQSFVHIVQMFINTS |

**Full sequences**

**>Pembrolizumab-IL-15Rα-IL-15 light chain**

MGWSCIILFLVATATGVHSDVQLLEEIVLTQSPATLSLSPGERATLSCRASKGVSTSGYSYLHWYQQKPGQAPRLLIYLASYLESGVPARFSGSGSGTDFTLTISSLEPEDFAVYYCQHSRDLPLTFGGGTKVEIKRTVAAPSVFIFPPSDEQLKSGTASVVCLLNNFYPREAKVQWKVDNALQSGNSQESVTEQDSKDSTYSLSSTLTLSKADYEKHKVYACEVTHQGLSSPVTKSFNRGEC

**> Pembrolizumab-IL-15Rα-IL-15 heavy chain**

MGWSCIILFLVATATGVHSDVQLLEQVQLVQSGVEVKKPGASVKVSCKASGYTFTNYYMYWVRQAPGQGLEWMGGINPSNGGTNFNEKFKNRVTLTTDSSTTTAYMELKSLQFDDTAVYYCARRDYRFDMGFDYWGQGTTVTVSSASTKGPSVFPLAPCSRSTSESTAALGCLVKDYFPEPVTVSWNSGALTSGVHTFPAVLQSSGLYSLSSVVTVPSSSLGTKTYTCNVDHKPSNTKVDKRVESKYGPPCPPCPAPEFLGGPSVFLFPPKPKDTLMISRTPEVTCVVVDVSQEDPEVQFNWYVDGVEVHNAKTKPREEQFNSTYRVVSVLTVLHQDWLNGKEYKCKVSNKGLPSSIEKTISKAKGQPREPQVYTLPPSQEEMTKNQVSLTCLVKGFYPSDIAVEWESNGQPENNYKTTPPVLDSDGSFFLYSRLTVDKSRWQEGNVFSCSVMHEALHNHYTQKSLSLSLGKGGGGSGGGGSGGGGSITCPPPMSVEHADIWVKSYSLYSRERYICNSGFKRKAGTSSLTECVLNKATNVAHWTTPSLKCIRGGGGSGGGGSGGGGSGGGGSNWVNVISDLKKIEDLIQSMHIDATLYTESDVHPSCKVTAMKCFLLELQVISLESGDASIHDTVENLIILANNSLSSNGNVTESGCKECEELEEKNIKEFLQSFVHIVQMFINTS
