# Supplementary material for: Plant-derived Pembrolizumab in conjugation with IL-15Rα-IL-15 complex shows effective anti-tumor activity
Source: PLoS One. 2025 Jan 14;20(1):e0316790. doi: 10.1371/journal.pone.0316790 (PMC11731737; doi:10.1371/journal.pone.0316790)
Supplement: S2 Table — (DOCX) [file pone.0316790.s002.docx]

**S2 Table.** Mean absorbance of PD-1 binding data and statistical comparison between Pembrolizumab-IL-15Rα-IL15 versus Keytruda.

| **Concentration (µg/mL)** | **Average Abs 450 nm** | | **P value**  **(vs. Keytruda)** |
| --- | --- | --- | --- |
|  | **Pembrolizumab-IL-15Rα-IL15** | **Keytruda** |  |
| 0.002441 | 0.1765 | 0.2045 | >0.9999 |
| 0.009766 | 0.2390 | 0.2780 | >0.9999 |
| 0.039060 | 0.3310 | 0.4735 | 0.7750 |
| 0.156300 | 0.8525 | 1.2495 | 0.0237 * |
| 0.625000 | 1.6125 | 1.8660 | 0.2022 |
| 2.500000 | 2.0035 | 1.973 | >0.9999 |
| 10.000000 | 1.9590 | 1.845 | 0.9123 |
| 40.000000 | 2.0520 | 1.899 | 0.7123 |

* P-value < 0.05
