# Supplementary material for: Plant-derived Pembrolizumab in conjugation with IL-15Rα-IL-15 complex shows effective anti-tumor activity
Source: PLoS One. 2025 Jan 14;20(1):e0316790. doi: 10.1371/journal.pone.0316790 (PMC11731737; doi:10.1371/journal.pone.0316790)
Supplement: S3 Table — (DOCX) [file pone.0316790.s003.docx]

**S3 Table.** Post-translational modifications of pembrolizumab-IL-15Rα-IL-15 detected with LC-MS peptide mapping analysis.

| **Modification** | **Part** | **Position** | **% modified** |
| --- | --- | --- | --- |
| Oxidation | Pembrolizumab heavy chain | M87 | 0.56 |
| Oxidation | Pembrolizumab heavy chain | M111 | 35.05 |
| Oxidation | Pembrolizumab heavy chain | M258 | 52.19 |
| Oxidation | Pembrolizumab heavy chain | M364 | 6.32 |
| Oxidation | Pembrolizumab heavy chain | M434 | 14.36 |
| Oxidation | IL-15Rα | M475 | 21.37 |
| Oxidation | IL-15 | M572 | 19.56 |
| Oxidation | IL-15 | M662 | 25.13 |
| Phosphorylation | Pembrolizumab light chain | S213 | 0.45 |
| Phosphorylation | Pembrolizumab heavy chain | S389 | 2.89 |
| Phosphorylation | Pembrolizumab heavy chain | T470 | 2.54 |
| Phosphorylation | Pembrolizumab heavy chain | S604 | 26.10 |
| Phosphorylation | Pembrolizumab heavy chain | T615 | 17.40 |

M: methionine, S: serine, T: threonine
