# Supplementary material for: Plant-derived Pembrolizumab in conjugation with IL-15Rα-IL-15 complex shows effective anti-tumor activity
Source: PLoS One. 2025 Jan 14;20(1):e0316790. doi: 10.1371/journal.pone.0316790 (PMC11731737; doi:10.1371/journal.pone.0316790)
Supplement: S1 Fig — The cropped areas are indicated in red. (DOCX) [file pone.0316790.s004.docx]

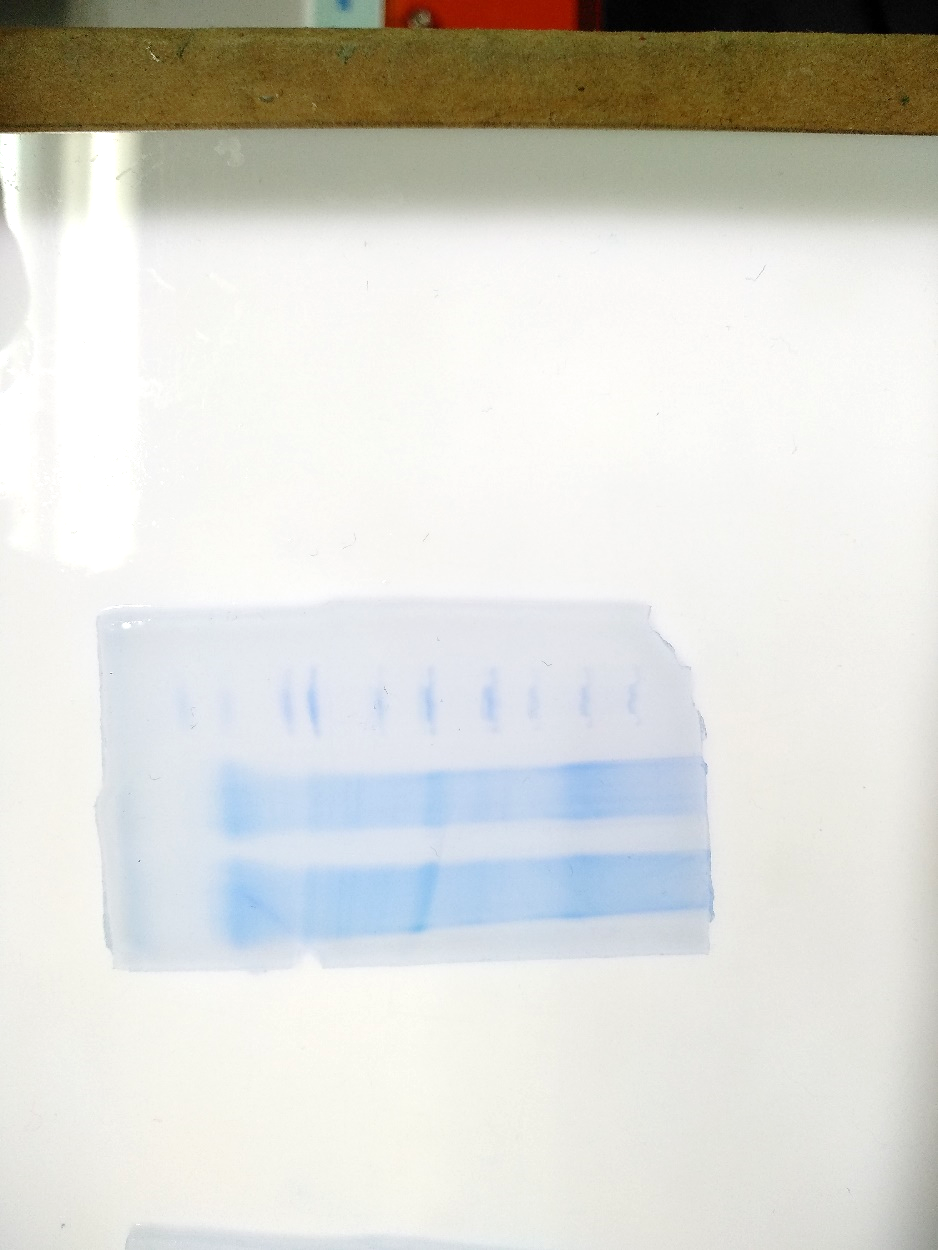

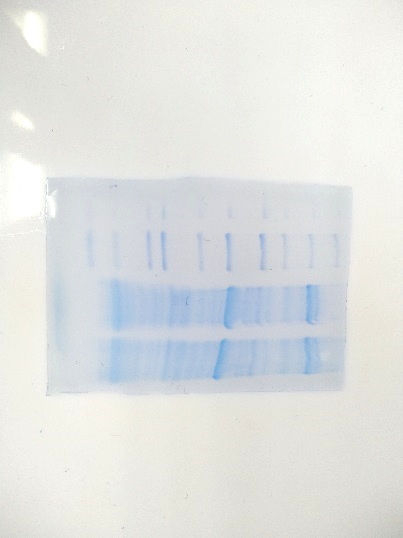


**B**

**A**

Figure 2D

Figure 2C

**S1** **Fig.** Raw files of SDS-PAGE gels shown in Figure 2C and 2D. The cropped areas are indicated in red.
