# Supplementary material for: Plant-derived Pembrolizumab in conjugation with IL-15Rα-IL-15 complex shows effective anti-tumor activity
Source: PLoS One. 2025 Jan 14;20(1):e0316790. doi: 10.1371/journal.pone.0316790 (PMC11731737; doi:10.1371/journal.pone.0316790)
Supplement: S2 Fig — The cropped areas are indicated in red. (DOCX) [file pone.0316790.s005.docx]

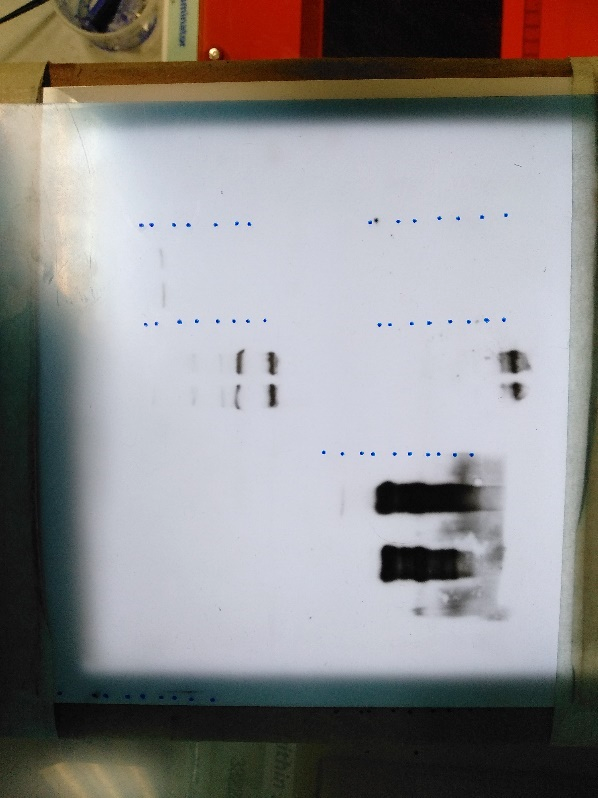


**D**

**C**

**A**

**B**

Figure 2H

Figure 2G

Figure 2F

Figure 2E

**S2 Fig.** Raw file of Western blot films shown in Figure 2E–H. The cropped areas are indicated in red.
