# Supplementary material for: Plant-derived Pembrolizumab in conjugation with IL-15Rα-IL-15 complex shows effective anti-tumor activity
Source: PLoS One. 2025 Jan 14;20(1):e0316790. doi: 10.1371/journal.pone.0316790 (PMC11731737; doi:10.1371/journal.pone.0316790)
Supplement: S4 Fig — (A) Deconvulated peak of pembrolizumab light chain. Protein peak was observed at 24.4 kDa with an inset showing MS spectrum from 500–3,000 m/z. (B) Deconvulated peak of pembrolizumab-IL-15Rα-IL-15 heavy chain. Protein peak was observed at 73.3 kDa with an inset showing MS spectrum from 800–3,000 m/z. (DOCX) [file pone.0316790.s007.docx]

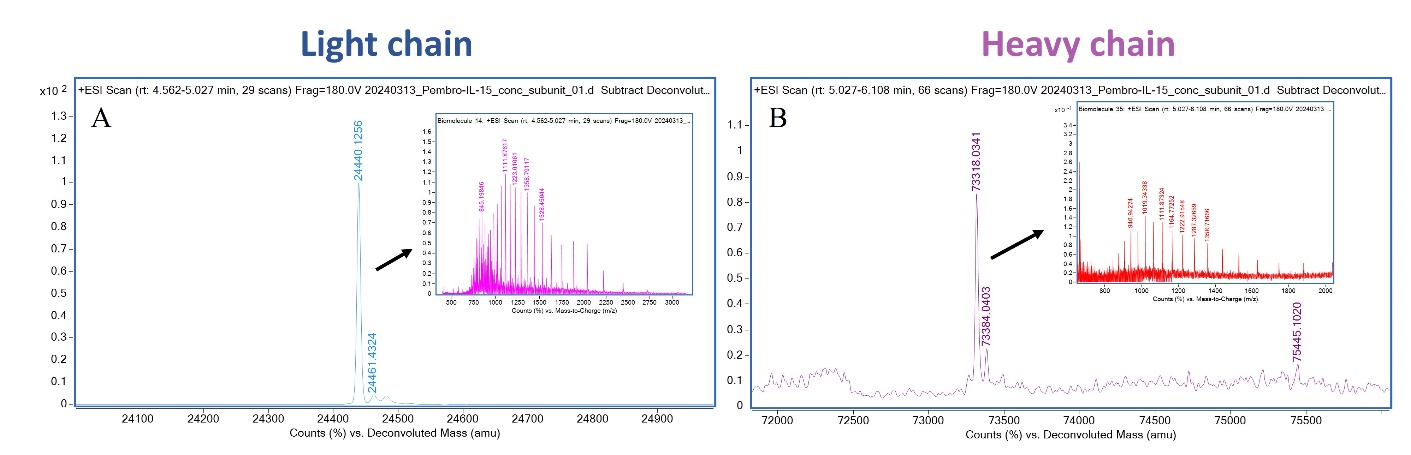


**S4** **Fig.** Subunit mass analysis of pembrolizumab-IL-15Rα-IL-15 using LC-MS. (A) Deconvulated peak of pembrolizumab light chain. Protein peak was observed at 24.4 kDa with an inset showing MS spectrum from 500–3,000 m/z. (B) Deconvulated peak of pembrolizumab-IL-15Rα-IL-15 heavy chain. Protein peak was observed at 73.3 kDa with an inset showing MS spectrum from 800–3,000 m/z.
