# Supplementary material for: Plant-derived Pembrolizumab in conjugation with IL-15Rα-IL-15 complex shows effective anti-tumor activity
Source: PLoS One. 2025 Jan 14;20(1):e0316790. doi: 10.1371/journal.pone.0316790 (PMC11731737; doi:10.1371/journal.pone.0316790)
Supplement: S5 Fig — Chromatogram of specific peptide showing non-glycosylated and GnGnXF-glycosylated peaks (A). Non-glycosylated peak was eluted at 60.9 min, while glycosylated peaks were eluted at 60.2 min. MS spectrum of GnXF and GnGnXF glycosylated mass from 1490–1600 m/z (B). Inset table summarizing N-glycosylation data, where 94.45% of the molecule was not glycosylated. (DOCX) [file pone.0316790.s008.docx]

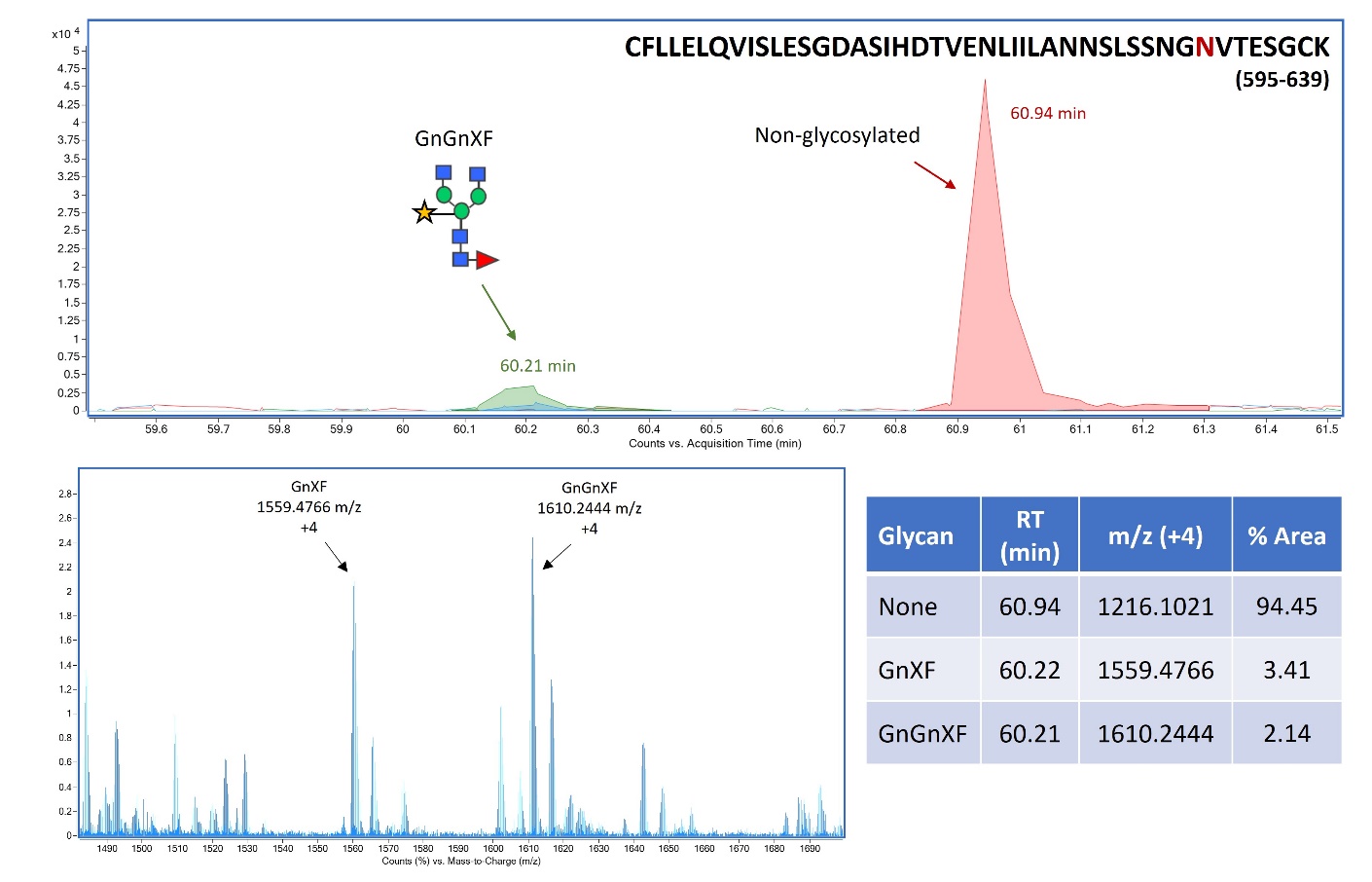


**S5 Fig.** *N*-glycosylation analysis at N632 position of pembrolizumab-IL-15Rα-IL-15 heavy chain. Chromatogram of specific peptide showing non-glycosylated and GnGnXF-glycosylated peaks (A). Non-glycosylated peak was eluted at 60.9 min, while glycosylated peaks were eluted at 60.2 min. MS spectrum of GnXF and GnGnXF glycosylated mass from 1490–1600 m/z (B). Inset table summarizing *N*-glycosylation data, where 94.45% of the molecule was not glycosylated.
