# Supplementary figures and images for: Plant-derived Pembrolizumab in conjugation with IL-15Rα-IL-15 complex shows effective anti-tumor activity
Source: PLoS One. 2025 Jan 14;20(1):e0316790. doi: 10.1371/journal.pone.0316790 (PMC11731737; doi:10.1371/journal.pone.0316790)

**Figure 2 C**

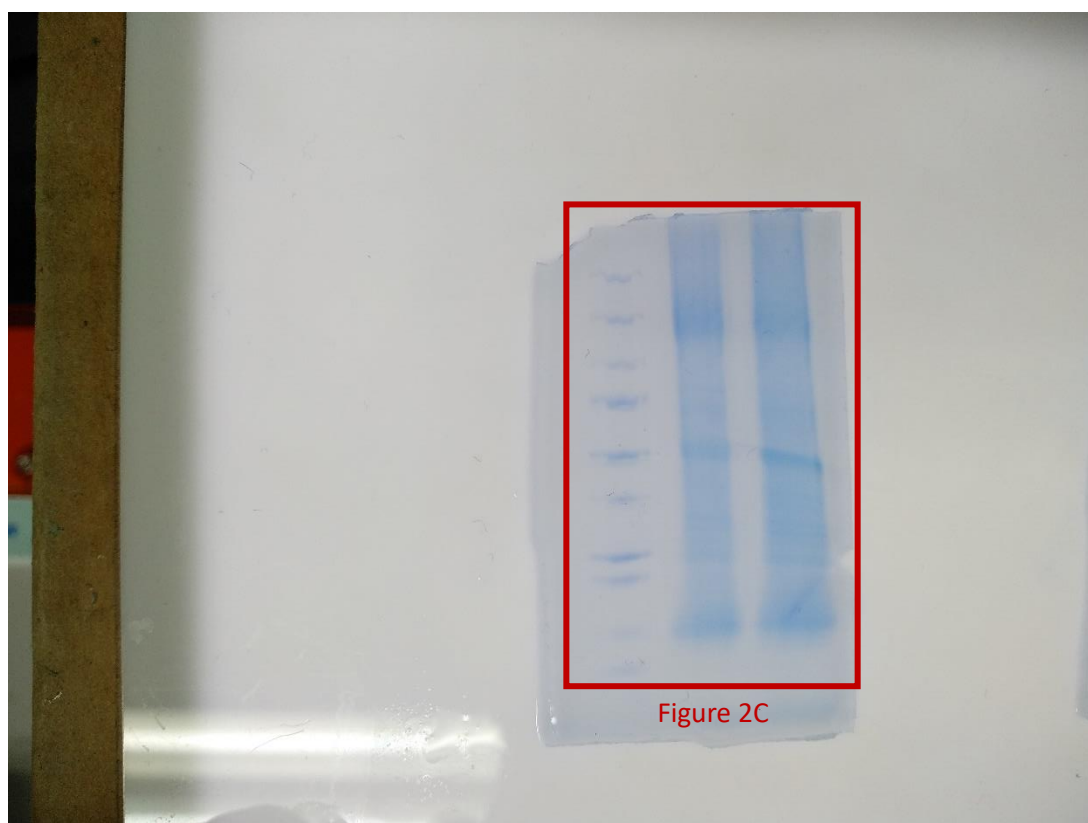

**Figure 2D**

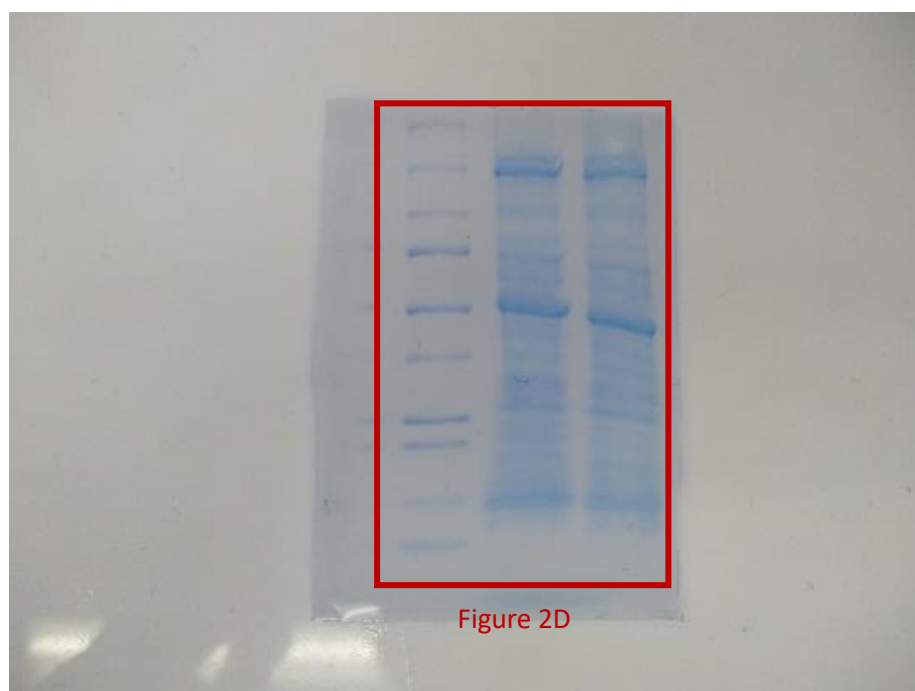

**Figure 2 E-F-G-H**

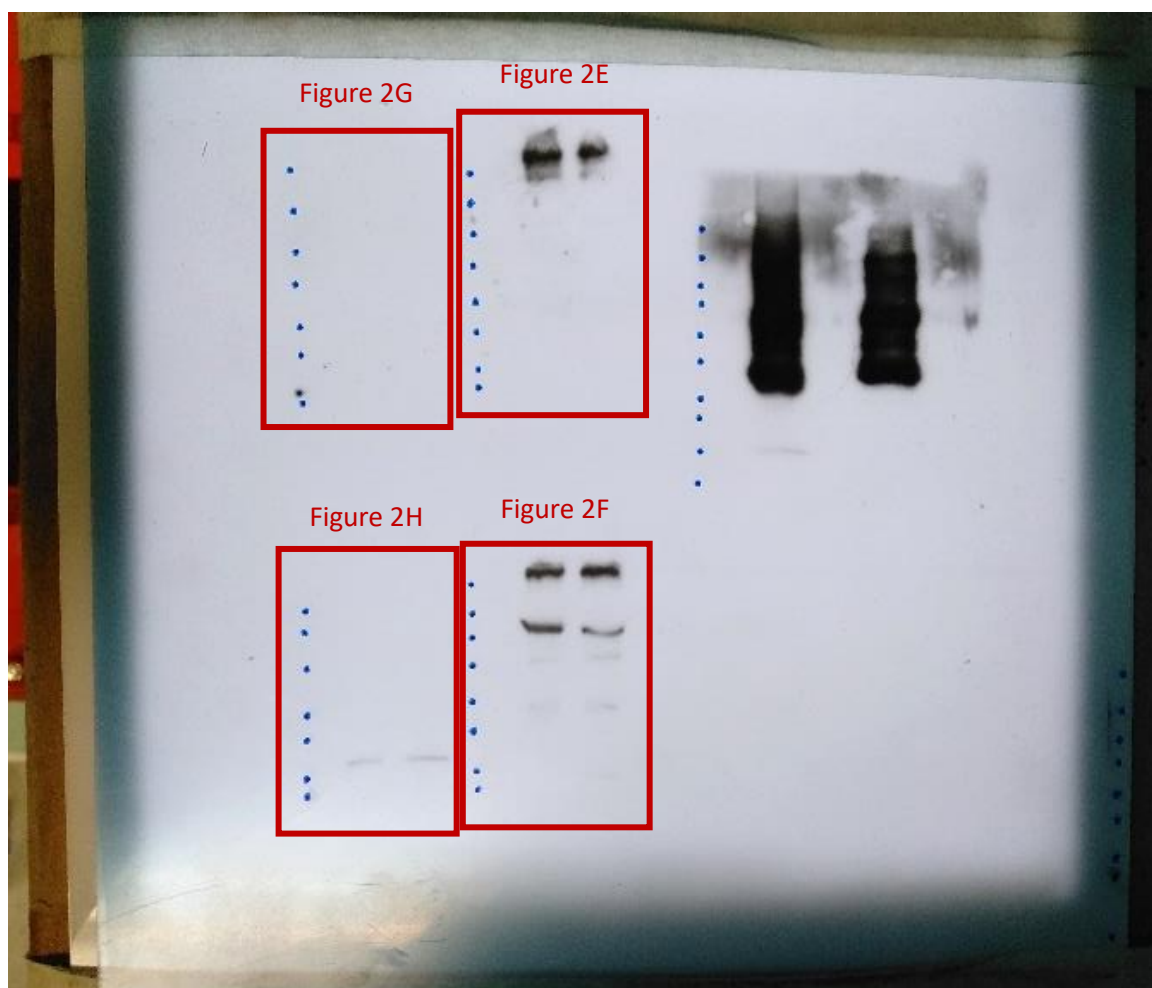

**Figure 3A**

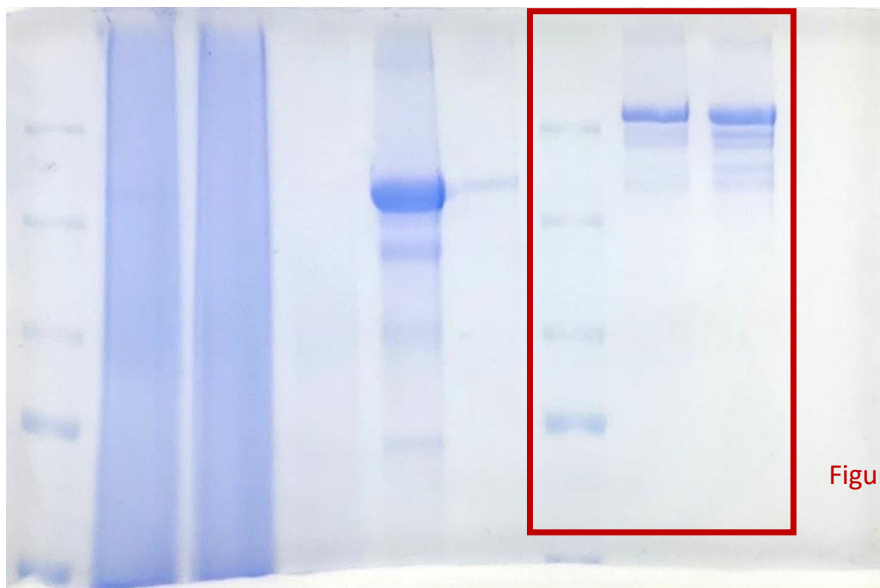

Figure 3A

**Figure 3B**

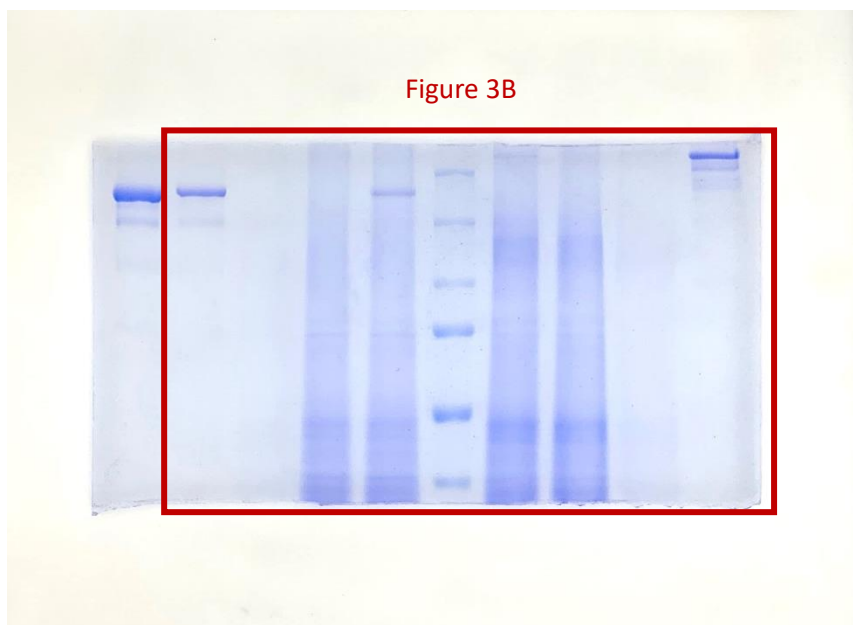

Figure 3B

Supplement: S1 Raw images — (PDF) [file pone.0316790.s009.pdf]
